# Supplementary material for: Evolve with your research: stepwise system evolution from document-driven to fact-centric research data management in materials science
Source: J Cheminform. 2026 Mar 17;18:44. doi: 10.1186/s13321-026-01180-y (PMC13063433; doi:10.1186/s13321-026-01180-y)
Supplement: Supplementary file 1 — Additional file 1. [file 13321_2026_1180_MOESM1_ESM.docx]

Supplementary Information for “Evolve with Your Research – Stepwise System Evolution from Document-driven to Fact-centric Research Data Management in Materials Science”

MatInf results in the FAIR Data Self-Assessment Tool

Using the *FAIR Data Self-Assessment Tool* [ARDC; <https://ardc.edu.au/resource/fair-data-self-assessment-tool/>], and answering all questions to the best of our knowledge, we obtained an overall **FAIRness score of 82%** for the evaluated CRC/TRR 247 MatInf tenant, available at <https://crc247.mdi.ruhr-uni-bochum.de/>.

The Supplementary Material provides screenshots of the complete, point-by-point evaluation. Each screenshot documents the individual assessment questions, the available answer options, and the selected responses that together lead to the final FAIRness score.


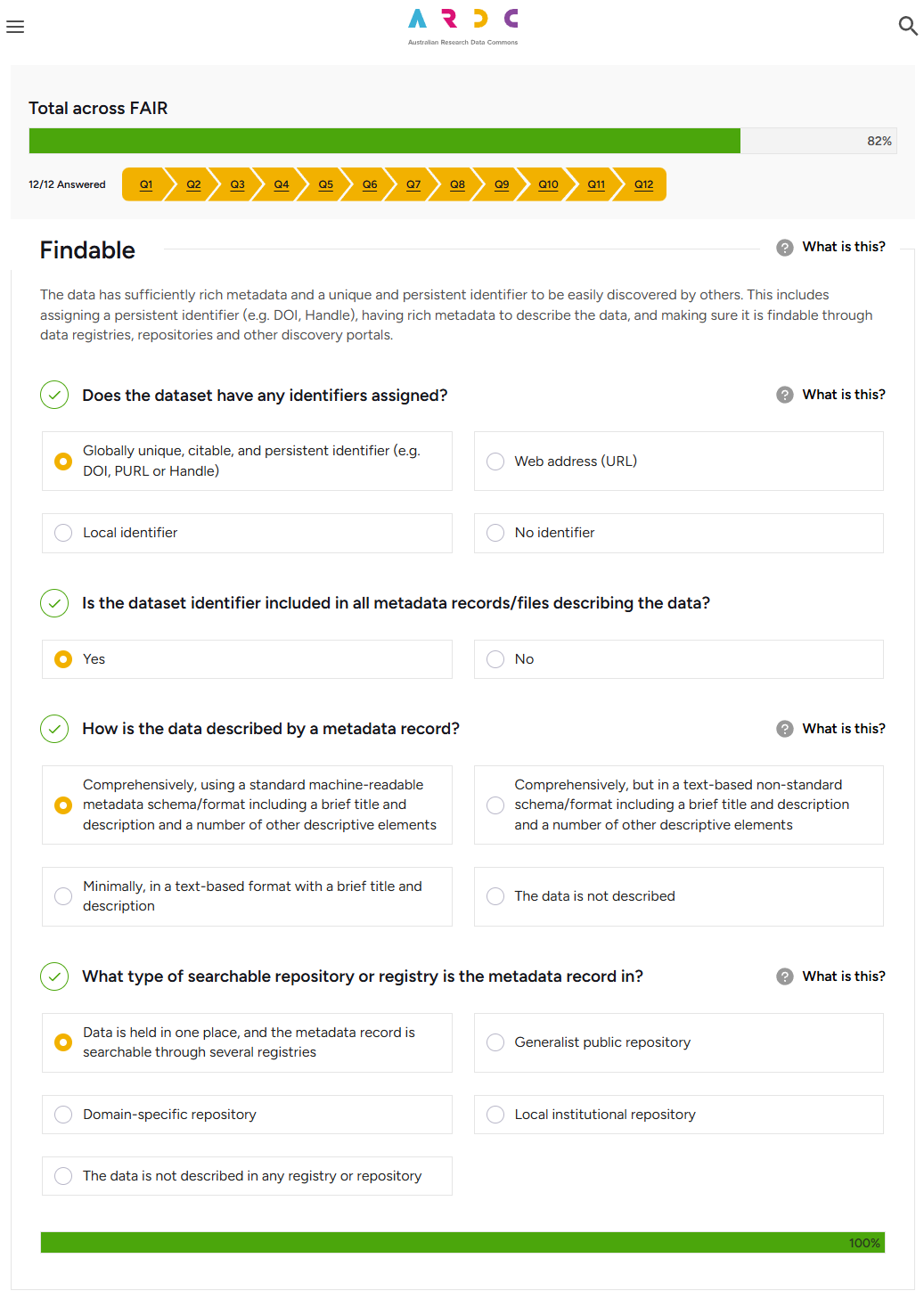


Supplementary Figure 1. Evaluation of the *Findable* principle for the CRC/TRR 247 MatInf tenant.


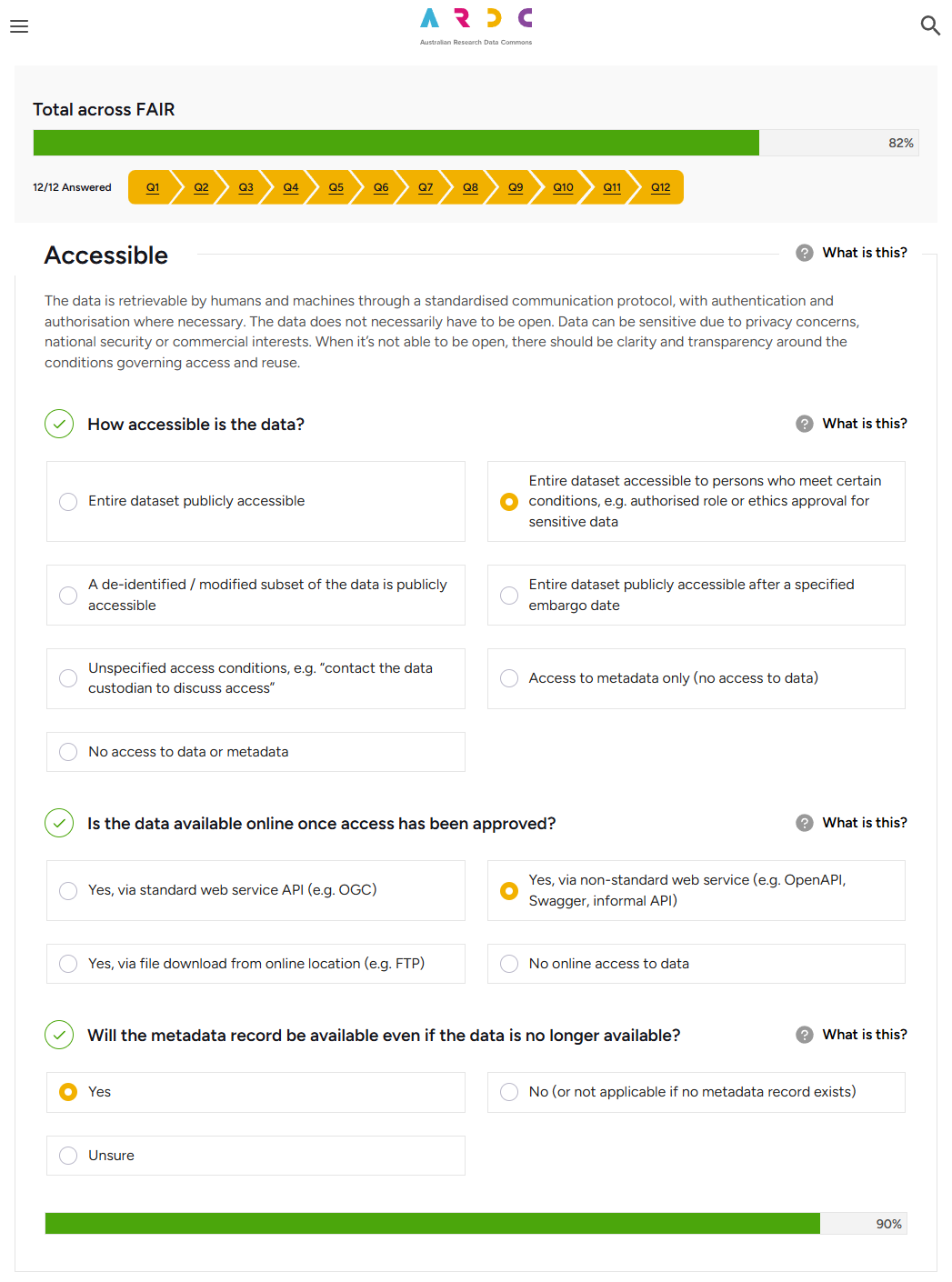


Supplementary Figure 2. Evaluation of the *Accessible* principle for the CRC/TRR 247 MatInf tenant.


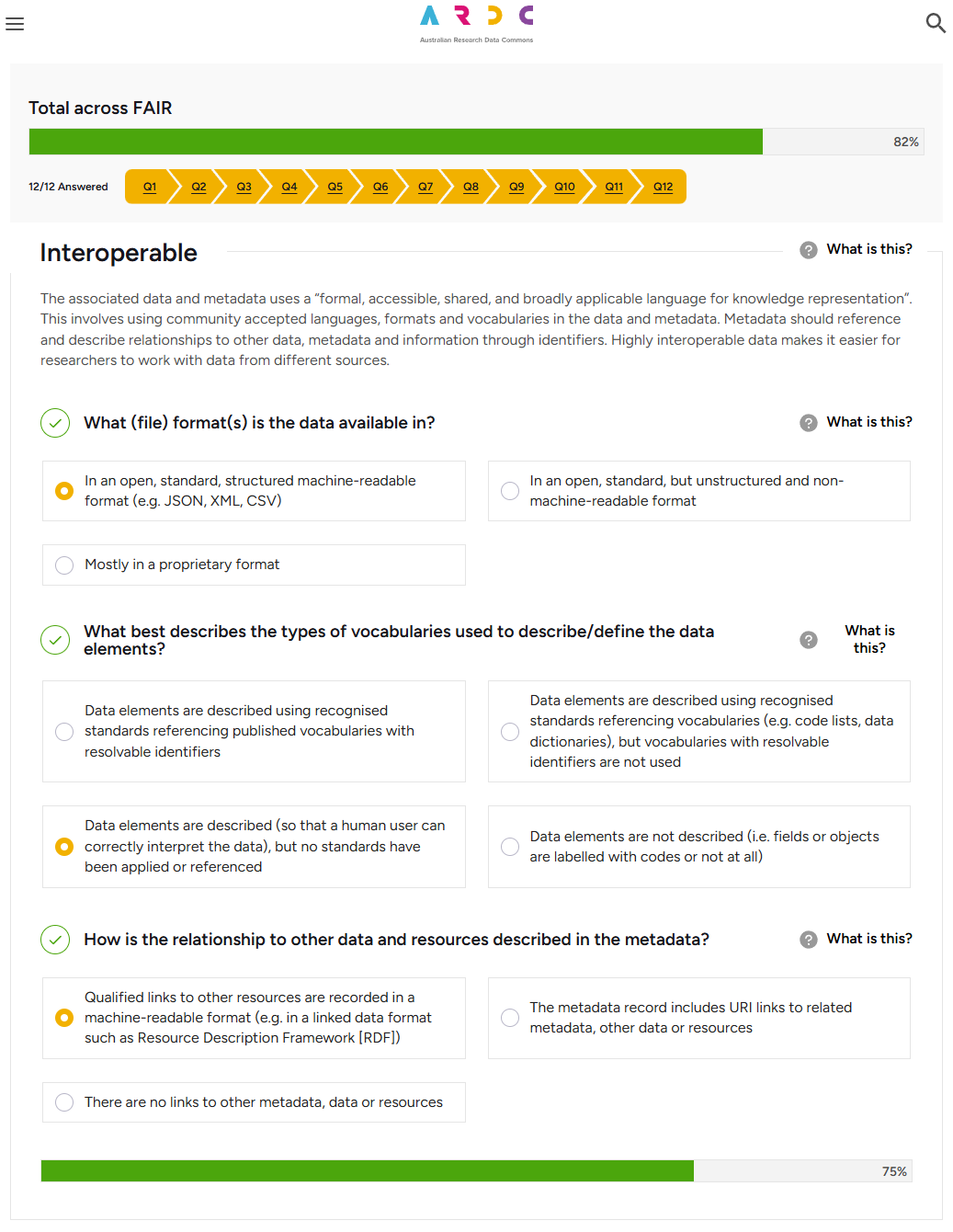


Supplementary Figure 3. Evaluation of the *Interoperable* principle for the CRC/TRR 247 MatInf tenant.


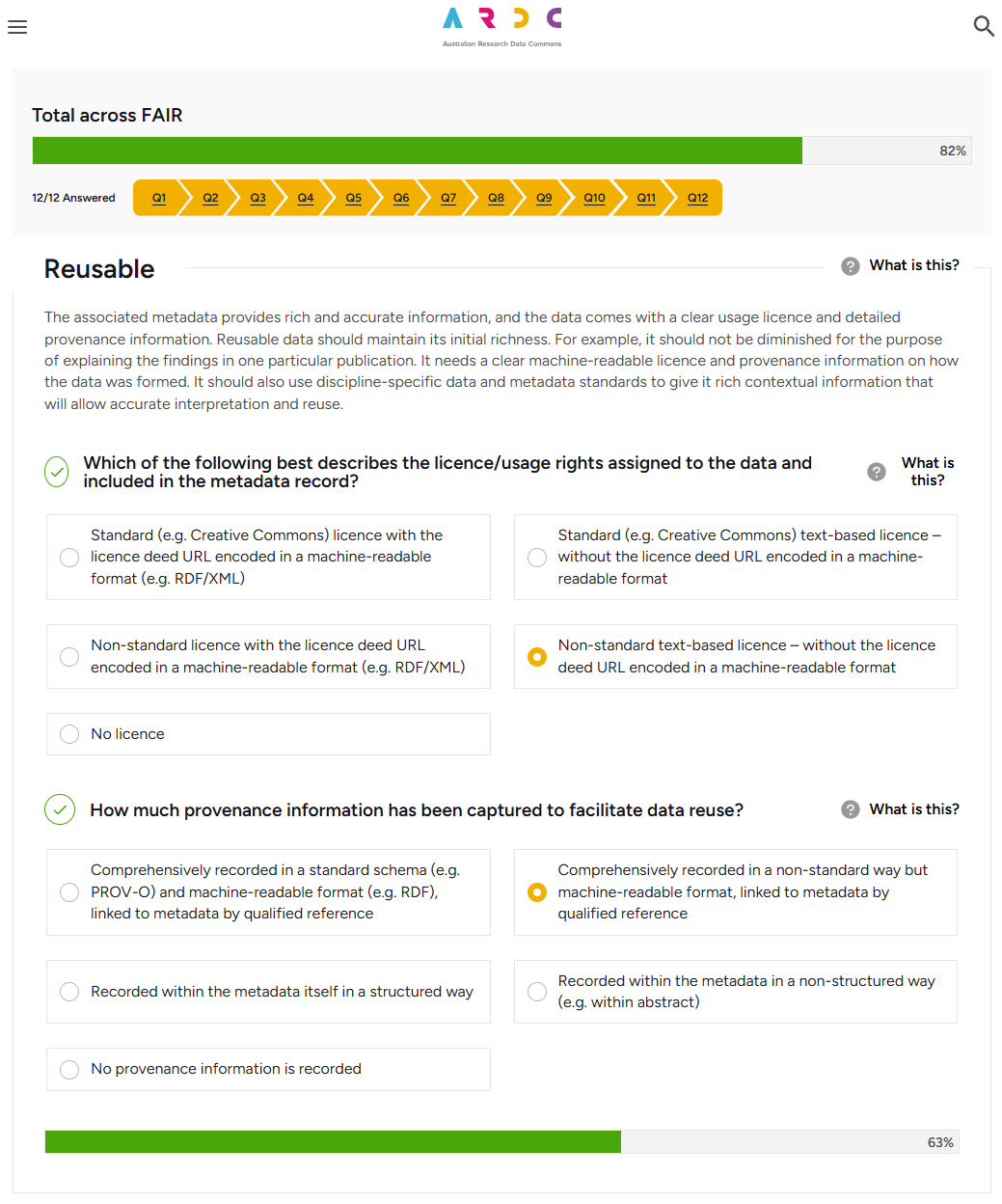


Supplementary Figure 4. Evaluation of the *Reusable* principle for the CRC/TRR 247 MatInf tenant.
